# A South African Epidemiological Study of Fatal Drownings: 2016–2021

**DOI:** 10.3390/ijerph192215121

**Published:** 2022-11-16

**Authors:** Jill Fortuin, Innocent Karangwa, Nongcebo Mahlalela, Cleeve Robertson

**Affiliations:** 1 National Sea Rescue Institute, Cape Town 7441, South Africa; nongcebo@searescue.org.za (N.M.); cleeve@searescue.org.za (C.R.); 2 Department of Human Biology, Faculty of Health Sciences, University of Cape Town, Cape Town 7701, South Africa; 3 Department of Statistical Science, University of Cape Town, Cape Town 7701, South Africa; innocent.karangwa@uct.ac.za

**Keywords:** drowning, epidemiology, low and middle income, South Africa

## Abstract

Drowning is a serious public health concern. Low-and-middle-income countries are the most affected by drowning, as they carry 90% of the global drowning burden. The purpose of this retrospective epidemiological study is to provide an overview of fatal drownings in South Africa between 2016 and 2021. The data used for the study were obtained from the South African Police Service. Descriptive statistics were used to summarize the data. Statistical analyses included a t-test and chi-square test. The results indicate that the average number of fatal drownings per annum is 1477 in South Africa, with an average drowning rate of 2.54 per 100,000 population for the period 2016 to 2021. The KwaZulu-Natal province had the highest incidence of drowning. The 0–4-year-age group has the highest prevalence of drowning among all the age categories. More males drowned in South Africa compared to females.

## 1. Introduction

In recent years, research into fatal drownings has increased, highlighting fatal drownings as a serious public health concern [1]. The World Health Organization (WHO), together with various organizations, has been at the forefront of advocating for drowning prevention interventions [2]. However, for drowning prevention interventions to be targeted, this requires guidance from epidemiological data [3]. Most epidemiological studies have taken place in developed countries, and as such, the recommended drowning prevention interventions are designed for this target population and may not be applicable to low- and middle-income countries (LMICs) [4]. 

LMICs have been referred to as having the highest burden of global drownings [5]. Approximately 90% of the annual global drownings occur in LMICs [6,7,8]. Furthermore, Africa has been reported to have the highest drowning rates [5]. Varying reasons have been cited as contributing factors for the high burden of fatal drownings in LMICs, which include, but are not limited to, the lack of drowning prevention initiatives, the lack of regulations and policies related to drowning prevention, insufficient water safety awareness and education campaigns, and a lack of basic swimming skills [6,9,10]. 

The key variables identified in various studies pertaining to fatal drownings include age, sex, location, day, and time of fatal drowning incidents [6,11]. Ethnicity is an important variable in South Africa, as it provides crucial information in terms of risk factors affecting a particular ethnic group [12]. 

In LMICs, fatal drowning incidents are prevalent in children in the age group under 5 years old. A report in 2013 indicated that 50% of drownings occur in children between 0 and 4 years [13]. In Bangladesh, 43% of drowning deaths occur in children between 12 and 59 months [7]. Often, drownings among children 0–4-years-old happen within 3 to 20 m of the home and where there was no adult supervision [8,14]. 

Several studies indicate that fatal drownings are more prevalent in males than females [6,13,15]. Numerous reasons have been cited in connection with why males are more likely to drown; these include increased exposure to water, males being more confident about their swimming ability than females, cultural bias, and males being more likely to take risks [6,16].

Several researchers have also categorized drownings as occurring in either small or large bodies of water; the findings of these studies were that most drownings occur in small bodies of water [6]. Freshwater has been reported as a more prevalent site for fatal drownings [17]. LMICs have a higher incidence of freshwater drownings, especially since freshwater drownings often include cisterns, wells, and small bodies of water [6]. For instance, freshwater drownings in Bangladesh account for 80% of all drownings [18]. The highest fatal drowning incidence in Australia was reported in natural-water locations [19]. 

Moreover, there has been an increase in drowning prevention research. In 1995, contributions to drowning research were represented by just nine countries, but in the period from 2015 to 2020, 79 countries were represented in drowning research [1]. In the list of the top ten countries contributing to drowning research, African countries do not appear, and only two LMICs appear in the top ten [1]. A literature review indicates that of the sixteen peer-reviewed drowning publications, eleven articles are from South Africa [20]. These publications in South Africa are limited to particular regions in terms of drowning epidemiology, which therefore necessitates increasing the country’s epidemiological study of fatal drownings.

The rationale for undertaking this study is to understand the fatal drowning epidemiology in South Africa and to develop appropriate drowning prevention interventions in response to the findings. This retrospective epidemiological study aims to provide a comprehensive overview of fatal drownings in South Africa between 2016 and 2021. 

## 2. Materials and Methods

This six-year retrospective epidemiological study was conducted to explore fatal drowning incidents in South Africa between 1 January 2016 and 31 December 2021. Fatal drowning was defined as respiratory impairment from immersion or submersion that results in death [3]. 

A death in South Africa is reported using a Death Report Form (Form BI–1680), which is often completed by a medical officer and/or the South African Police Services (SAPS) [21]. Since drowning is classified as an unintentional injury or unnatural death, it will be reported to SAPS, who will open a case for further investigation. 

The SAPS has given us approval for the use of their recorded drowning data for the period from 2016 to 2021. All identifying information for the data received was omitted; this included unique SAPS case numbers, names, and surnames of the deceased. In addition, ethical approval was received from Pharma Ethics, an independent organization awarding ethical approval to non-academic organizations such as the National Sea Rescue Institute. 

The drowning data available included age; ethnicity; sex; location of drowning; circumstance of drowning; and the date, day, and time of the drowning incidence. Descriptive statistics were used to summarize data, and the results were presented using frequency tables and graphs. Drowning mortality rates and age-standardized drowning rates per 100,000 population were calculated. 

Age-standardized drowning mortality rates (per 100,000 population) for sex (males and females) and ethnic groups (Blacks, Coloureds, Indians, and Whites) were calculated. The 2021 South African mid-year population estimates were used to calculate all age-adjusted rates [22]. The t-test and chi-square were used to investigate whether drowning fatalities differed between males and females and across ethnic groups, respectively.

## 3. Results

In Table 1, the number of drowning incidents per year for the 2016 to 2021 period is presented, with the highest incidence of drownings being recorded as 1526 in 2021 and the average drowning per annum for the period being 1477. 

### 3.1. Location and Timing

**Figure 1:**
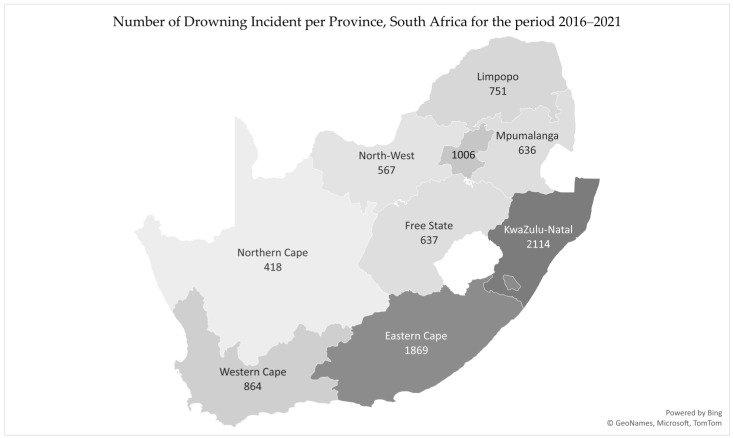
Number of drowning incidents per province, 2016–2021.

The KwaZulu-Natal province has the most reported drownings, with 2114 drownings recorded from 2016 to 2021, followed by the Eastern Cape, Gauteng, and the Western Cape; this distribution is depicted in Figure 1. 

**Figure 2:**
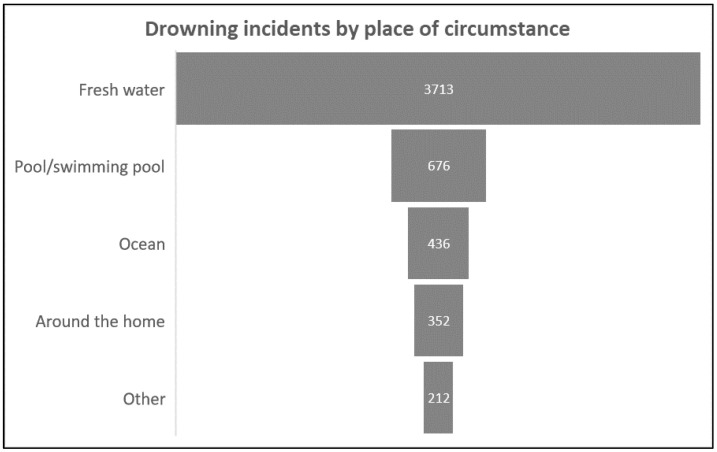
Drowning incidence by place of circumstance in South Africa, 2016–2021.

The place and circumstance of the drownings were categorized into freshwater (which includes canals, dams, dipping tanks, ditches, dongas, gully holes, lagoons, mineshafts, reservoirs, rivers, stormwater pipes, streams, swamps, wells, etc.), pools or swimming pools, the ocean (which includes harbours, sea or salt water), around the home (which includes baths, buckets of water, drums of water, fish ponds, fountains, toilets, septic tanks), and others (where drowning location is not specified). The highest number of drownings occurred in freshwater, with 3713 drownings reported from 2016 to 2021; this is illustrated in Figure 2. 

During the month of January, the number of fatal drownings was recorded as 1171, whereas February recorded 931 and November recorded 882. Sundays were the day of the week when fatal drownings (*n* = 1591) occurred more frequently, followed by Saturdays (*n* = 1503). Fatal drownings have been most frequently reported as occurring between 16:00 and 19:59 (*n* = 2639).

### 3.2. Sex and Age

The results indicate that fatal drowning incidents are the highest among males, who constituted 5820 drownings between 2016 and 2021, whereas only 1357 females drowned during that period.

Table 2 illustrates the sex- and age-specific drowning rates in South Africa between 2016 and 2021. Males had the highest drowning rate (per 100,000 population) in all age groups, with the highest age-specific drowning rate being 56 males per 100,000 population among the 0–4 year-old age group from 2016 to 2021.

For the 2016 to 2021 period, the overall age-adjusted female drowning rate is four people per 100,000 population; for males, the overall age-adjusted male drowning rate is 17.8 people per 100,000 population. 

The age group wherein the most fatal drownings occurred from 2016 to 2021 was the 0–4 year-old age category, with 2755 incidents and an age-adjusted drowning rate of 48 per 100,000 population. The other age group wherein the fatal drowning incidence was high included the 5–9 years, 10–14 years, and 15–19 years old age groups, as shown in Table 2. The male: female ratio results (>1) indicate that drowning fatalities were higher in males than in females. The t-test indicates that there was a statistically significant difference in the proportions of drownings between males and females (*p*-values < 0.05).

Table 3 depicts the age- and ethnicity-specific drowning rates. The 0–4 year-old age category had the highest burden of fatal drownings among all the age groups. In particular, the white ethnic group in the 0–4 years old age category had the highest age- and ethnicity-specific drowning rate for the 2016–2021 period, which was 57 per 100,000 population. For the other ethnic groups in this age category, the drowning rates were 41 per 100,000 for the coloured ethnic group and 36 per 100,000 for the black ethnic group. 

The cumulative age-adjusted drowning rate per 100,000 population is 12.5 for the coloured ethnic category from 2016 to 2021. In comparison, the age-adjusted drowning rate per 100,000 population is 11.1 for the black ethnic category. The age-adjusted Indian ethnic group drowning rate per 100,000 population is 6.4. Lastly, the age-adjusted drowning rate per 100,000 population is 9 for the white ethnic category. 

The chi-square test (see Table 4) indicates that there was a statistically significant difference in the proportions of fatal drownings between Black, Coloured, White, and Indian ethnic groups (all *p*-values < 0.05). 

Table 5 presents the age-adjusted drowning rates in all South African provinces. As indicated, the highest age-adjusted drowning rate per province for the period 2016 to 2021 was reported for the Northern Cape at 34.4, followed by the Eastern Cape at 23.7, the Free State at 16.8, KwaZulu-Natal at 15.5, the Western Cape at 11.0, Limpopo at 10.1, Mpumalanga 10.0, North West at 8.6, and Gauteng being the lowest at 5.6.

To conclude, the crude drowning rate per 100,000 population in South Africa was 12.4 for the 2016 to 2021 period. Furthermore, the results indicate that, in all provinces, younger people are more likely to be involved in drowning incidents than older people.

## 4. Discussion

The average number of fatal drowning incidents that occurred in South Africa from 2016 to 2021 is 1477 drownings per year. This average is far below the average of other LMICs, where the average number of drownings have been reported as 4624 per annum [13]. South Africa is globally listed in the top 45 countries with the drowning rate of 4.06 per 100,000 population [23].

The results indicate that South Africa had an annual fatal drowning rate of 2.54 per 100,000 population from 2016 to 2021, with the highest drowning mortality rate reported in 2016 (2.7 per 100,000 population) and the lowest in 2020 (2.36 per 100,000 population). Although South Africa does not have as high of a drowning incidence as other LMICs, there remains a concern for the fatal drownings rates reported.

In 2021, 1526 fatal drownings were reported, which was the highest annual drowning incidence that occurred in the six-year period of reporting. When comparing the drownings that occurred in 2020 (1401 fatal drownings) to 2021 (1526 fatal drownings), the increase in drownings could be attributed to the easing of restrictions following the reduced burden of COVID-19. Similarly, the low drowning incidence in 2020 could be attributed to the stringent COVID-19 restrictions, which included a ban on alcohol, restricted access to beaches and public swimming facilities, and limitations on gatherings. This suggests that alcohol does play a role in fatal drowning, and this is confirmed by the available literature [15].

The results indicate that drownings are most prevalent in KwaZulu-Natal, with 2114 drownings over the six-year period. Factors that could contribute to the fatal drowning burden include the province being the largest in geographical size; having the most water-related environments; experiencing a sub-tropical climate, which allows for swimming throughout the year; and having the largest population size among the nine provinces [24,25]. This epidemiological information guides drowning prevention strategies and aids in the design and implementation of drowning prevention interventions to ensure they are specific to the geographical regions.

Fatal drownings occurred more frequently in freshwater in South Africa during the 2016 to 2021 period. This is in keeping with the findings of the epidemiology of drowning in other LMICs, which indicates that drownings are common in small bodies of water, such as cisterns and wells, as they are more commonly used than saltwater bodies [6].

The research findings highlight that fatal drownings are most likely to occur in January and February; these months are the warmer months in South Africa. Research has indicated that the drowning incidence was higher in warmer months due to the increase in water-related activities [15]. The day of the week reported as the day when fatal drownings frequently occur is Sunday, which is a recreational day when people can visit dams, beaches, swimming pools, etc. With these results, adequate drowning prevention interventions can be implemented. For example, increasing lifeguard coverage during the months of January and February, especially on Sundays, with a focus on the time period from 16:00 to 19:59.

In this study, males represent 81% of all fatal drownings from 2016 to 2021, and females represent 19%. The sex ratio indicates that males are more likely to drown than females, as the ratio is greater than 1. The t-test indicated that there was a statistically significant difference between the proportions of females and males who drowned (*p*-value < 0.05). Multiple studies have reported that males engage in riskier physical behavior than females; hence, drownings are more likely to occur. This epidemiological finding highlights targeted education and behavior modification interventions required for males.

The age-specific drowning mortality rate indicates the 0–4-year age group as being of the highest risk, with a cumulative drowning incidence of 2755 fatal drownings and a rate of 48 per 100,000. This can be further represented as one drowning per day among 0–4-year-olds. The hypothesis is that the black population in the 0–4-year age group has the highest drowning incidence. However, this study has highlighted using the data, provided that the highest drowning incidence occurs in the white population among the 0–4 year-old age group. The province where most of the drownings occurred in this age category was the Northern Cape. This is an alarming statistic, as drowning is a preventable injury. In addition, the Northern Cape is not an area that has been prioritized for targeted drowning prevention interventions.

The results of the study provided evidence that the black ethnic group in South Africa has the highest incidence of fatal drownings (*n* = 6102) cumulatively for all age groups compared to the other ethnic groups included in the study for the 2016 to 2021 period. Further to this, there is statistical significance in the fatal drownings across ethnic groups, with the Black ethnic group having the highest drowning rate across all population groups. This high incidence of drowning among previously disadvantaged ethnic groups is influenced by various social determinants such as poverty, education, and infrastructure. Similar differences in drownings among ethnic groups have also been reported in other countries, where black persons are 1.5 times more likely to drown than white persons [26,27]. The evidence from the study provides vital information in ensuring that drowning prevention interventions are prioritized among the most at-risk ethnic groups.

The information gathered from this epidemiological study will assist in informing drowning prevention initiatives and guide the development of regulations and policies that aid drowning prevention.

The drowning data obtained only included fatal drownings that were reported or when a body was recovered. The information does include unreported incidences or incidences where a body was not recovered [28]. The reporting limitations are further complicated by the lack of classification of drownings; this is a global challenge, since drownings related to natural disasters (e.g., floods) are not reported [29]. Stemming from this, no drowning prevention interventions are being implemented or prioritized.

## 5. Limitations

The data available did not include information regarding the use of alcohol, swimming ability, or whether there was adult supervision. It also did not include what was the pre-event to drowning and if any cardiopulmonary resuscitation was administered. Future studies should include all of these variables to determine whether they contribute to drowning fatalities in South Africa.

## 6. Conclusions

This six-year retrospective epidemiological study provides a detailed overview of drowning incidents in South Africa, wherein all nine provinces and age groups are included in the study. The data highlight key areas of concern that will inform South Africa’s drowning prevention initiatives.

The time and location of drownings is important, as there is a distinct need for targeted drowning prevention interventions. The most prevalent time when drownings occurred is on Sundays, during the time from 16:00 to 19:59. The province with the highest incidence of drowning is the KwaZulu-Natal province.

With regards to the age of fatal drownings, the 0–4-year-old age category among the white and coloured population, in specific regions in South Africa such as the Northern Cape, Free State, and Eastern Cape, requires the prioritization of drowning prevention interventions. Males in all age categories should be the focus of drowning prevention initiatives, which could include swimming skills programs, education in schools, and the promotion of personal flotation devices in water sports. Across all age categories, the black ethnic group had the highest incidence of drowning. Specific interventions relevant and appropriate to the at-risk age categories and ethnic population groups are required.

Recommendations from this study include the improvement in the collection of fatal and non-fatal drowning data. Such data would contribute significantly to the design, development, and implementation of drowning prevention interventions that are specific to South Africa. The drowning data would also allow various organizations to measure the impact of their drowning prevention interventions.

## Figures and Tables

**Table 1:** Drowning mortality rates per annum in South Africa, 2016–2021.

Year	Fatal Drownings	Population Size [22]	Drowning Mortality Rate per 100,000 Population
2016	1519	56,207,646	2.70
2017	1482	57,009,756	2.60
2018	1463	57,792,518	2.53
2019	1471	58,558,270	2.51
2020	1401	59,308,690	2.36
2021	1526	60,978,505	2.50
Average	1477	58,309,231	2.54

**Table 2:** Sex- and age-specific drowning rates in South Africa, 2016–2021.

		Age-Specific Drowning Rates (per 100,000 Population)				
Age	Number of Drowning Incidents	Males	Females	Total	Male: Female Ratio	*P*-Values
0–4	2755	56	39	36.9	1.4	<0.002
5–9	763	20	6	12.7	3.3	<0.0001
10–14	803	21	6	13.5	3.3	<0.0001
15–19	508	17	3	9.9	5.0	<0.0001
20–24	384	13	3	7.6	4.8	<0.0001
25–29	420	13	2	7.3	5.3	<0.0001
30–34	405	12	2	6.9	5.6	<0.0001
35–39	315	10	2	5.9	4.3	<0.0001
40–44	266	11	3	6.3	4.0	<0.0001
45–49	199	10	3	5.6	3.8	<0.0001
50–54	183	12	2	6.5	5.1	<0.0001
55–59	123	9	2	5.0	3.9	<0.0001
60–64	106	11	2	5.6	4.9	<0.0001
65–69	68	9	2	4.6	6.0	<0.0001
70–74	65	10	4	6.0	2.5	<0.0001
75–79	44	10	5	6.5	1.9	<0.0500
80+	38	11	4	5.9	2.9	<0.005

**Table 3:** Number of drownings per age category in South Africa, 2016–2021.

Age Group	Mid-Year Population Size (Thousands) [22]	Number of Drowning Incidents	Drowning Rates per 100,000
0–4	5,708,956	2755	48
5–9	5,663,296	763	13
10–14	5,671,023	803	14
15–19	4,909,941	508	10
20–24	4,739,305	384	8
25–29	5,324,134	420	8
30–34	5,630,643	405	7
35–39	4,985,251	315	6
40–44	3,881,731	266	7
45–49	3,254,138	199	6
50–54	2,625,390	183	7
55–59	2,243,823	123	5
60–64	1,815,810	106	6
65–69	1,422,604	68	5
70–74	1,024,345	65	6
75–79	647,265	44	7
80+	595,323	38	6
Total	60,142,978	7445	12

**Table 4:** Age-specific and ethnicity-specific drowning rates in South Africa, 2016–2021.

Age	Black	Coloured	Indian/Asian	White	*P*-Values
0–4	36	41	27	57	<0.0001
5–9	13	15	4	4	<0.0001
10–14	15	12	2	1	<0.0001
15–19	10	11	3	4	<0.0001
20–24	8	11	6	1	<0.0001
25–29	7	10	5	6	<0.0001
30–34	7	7	4	4	<0.0001
35–39	6	8	3	3	<0.0001
40–44	6	9	6	5	<0.0001
45–49	5	11	4	6	<0.0001
50–54	7	7	7	5	<0.0001
55–59	5	6	5	5	<0.0001
60–64	6	7	3	5	<0.0001
65–69	5	4	3	4	<0.0001
70–74	7	1	2	8	<0.0001
75–79	7	3	4	7	<0.0001
80+	9	2	4	5	<0.0001

**Table 5:** Age-specific drowning rates per 100,000 population for South African provinces, 2016–2021.

	Age-Specific Drowning Rates (per 100,000 Population)								
Age (Years)	EC	FS	GP	KZN	LP	MP	NC	NW	WC
0–4	62	92	33	49	37	52	141	32	44
5–9	30	11	4	17	10	12	34	8	12
10–14	30	16	5	17	10	11	40	8	10
15–19	19	12	2	15	11	7	28	6	8
20–24	14	12	2	11	8	5	18	8	11
25–29	20	8	2	11	7	2	27	10	8
30–34	16	9	2	11	7	4	28	6	6
35–39	17	6	2	8	6	5	21	6	4
40–44	19	4	2	10	6	3	19	5	7
45–49	17	6	2	10	4	3	11	4	6
50–54	20	8	1	13	6	2	22	4	4
55–59	16	4	2	8	4	2	4	5	4
60–64	13	5	2	10	6	1	10	4	5
65–69	16	3	1	7	1	5	9	1	4
70–74	16	4	2	10	6	4	8	2	4
75–79	15	9	1	11	6	0	0	2	8
80+	10	7	8	7	6	2	6	0	4
Total	23.7	16.8	5.6	15.5	10.1	10.0	34.4	8.6	11.0

## Data Availability

The data presented in this study are available on request from the corresponding author. The data are not publicly available due to the data belonging to the South African Police Service.
